# Sodium Laurate, a Novel Protease- and Mass Spectrometry-Compatible Detergent for Mass Spectrometry-Based Membrane Proteomics

**DOI:** 10.1371/journal.pone.0059779

**Published:** 2013-03-28

**Authors:** Yong Lin, Linju Huo, Zhonghua Liu, Jianglin Li, Yi Liu, Quanze He, Xianchun Wang, Songping Liang

**Affiliations:** 1 Key Laboratory of Protein Chemistry and Developmental Biology of National Education Committee, College of Life Sciences, Hunan Normal University, Changsha, P. R. China; 2 National Research Center of Engineering and Technology for Utilization of Botanical Functional Ingredients, Hunan Agricultural University, Changsha, P. R. China; George Washington University, United States of America

## Abstract

The hydrophobic nature of most membrane proteins severely complicates their extraction, proteolysis and identification. Although detergents can be used to enhance the solubility of the membrane proteins, it is often difficult for a detergent not only to have a strong ability to extract membrane proteins, but also to be compatible with the subsequent proteolysis and mass spectrometric analysis. In this study, we made evaluation on a novel application of sodium laurate (SL) to the shotgun analysis of membrane proteomes. SL was found not only to lyse the membranes and solubilize membrane proteins as efficiently as SDS, but also to be well compatible with trypsin and chymotrypsin. Furthermore, SL could be efficiently removed by phase transfer method from samples after acidification, thus ensuring not to interfere with the subsequent CapLC-MS/MS analysis of the proteolytic peptides of proteins. When SL was applied to assist the digestion and identification of a standard protein mixture containing bacteriorhodoposin and the proteins in rat liver plasma membrane-enriched fractions, it was found that, compared with other two representative enzyme- and MS-compatible detergents *Rapi*Gest SF (RGS) and sodium deoxycholate (SDC), SL exhibited obvious superiority in the identification of membrane proteins particularly those with high hydrophobicity and/or multiple transmembrane domains.

## Introduction

Membranes are critical components of cellular structure and play important roles in partitioning of organelles, providing defense against foreign molecules and external conditions that may damage or destroy the cell and so on [1]. The functions of membranes are essentially carried out by membrane proteins. Of the membrane proteins, plasma membrane (PM) proteins are of particular importance because they play important biological and pharmacological roles in regulating the exchange of material and energy between cells and their environments [2]–[4]. Despite the biological importance of membrane proteins especially PM proteins, their analyses have lagged behind that of soluble proteins and still present a great challenge mainly because of their highly hydrophobic nature and low abundance [1], [5], [6]. The poor solubility of most membrane proteins especially integral membrane proteins in water or aqueous buffers has limited their solubilization, extraction and enzymolysis. Therefore, how to prepare membrane protein samples for mass spectrometric analysis is a subject worthy of investigation [1], [6], [7].

To improve the extraction and solubilization of membrane proteins, some additives were added to the buffer, including detergents, chaotropes, aqueous-organic solvents, organic acids, etc [8]. Compared with other additives, the detergents can more efficiently dissolve and denature a wider range of proteins including membrane proteins. Of the commonly used detergents, SDS has the strongest ability to disrupt biological membranes and extract the membrane proteins [9]. However, SDS is not compatible with protease activity and mass spectrometry (MS), and thus is limited in the application to membrane proteomics [10], [11]. Unlike SDS, an acid-labile surfactant (trade name *Rapi*Gest SF, RGS) solubilizes proteins without inhibiting enzyme trypsin or other common endopeptidases activity, and dose not interfere with mass spectrometric analysis because it degrades rapidly at low-pH conditions. Therefore, it was defined as an enzyme-friendly, MS-compatible detergent [10]. However, RGS is synthesized by Waters Corp., and, as the first commercially marketed enzyme activity- and MS-compatible detergent, is expensive, which limits its extensive application to membrane proteomics. Another typical enzyme activity- and MS-compatible detergent is sodium deoxycholate (SDC). SDC is compatible with tryptic digestion at concentrations up to 5.0% [12], Furthermore, SDC precipitates at low pH and can be removed from the tryptic digest sample by centrifugation or phase transfer following acidification, which eliminates its interference with mass spectrometric analysis [13]. In addition, compared with RGS, SDC is cheaper and easier to obtain. However, the main shortcoming of SDC is that its ability to lyse membranes and extract the membrane proteins is weaker than that of SDS.

Therefore, it is a significant work to seek an additive that is more suitable for the extraction and identification of membrane proteins. Starting with the comparatively analyzing the structures of SDS and SDC, we have made a series of detergent screenings and speculated that sodium laurate (SL) should have strong potential to improve the shotgun analysis of membrane proteome, because it has an typical linear head and tail structure with a long hydrophobic hydrocarbon chain similar to that of SDS and a hydrophilic head (a carboxyl group) similar to that of SDC, which is favourable for the removal of it from the sample by acidification before LC-MS/MS.

In the present study, through a series of comparative studies, we demonstrated that sodium laurate (SL) not only had strong ability to lyse membranes and extract membrane proteins, but also could enhance enzyme activity when its concentration was 0.1%. Furthermore, SL was found to be easily removed by phase transfer method from the sample, thus avoiding the possible interference with the CapLC-MS/MS analysis of proteolytic peptides of proteins. Finally, the applicability of SL was demonstrated using standard proteins and rat liver plasma membrane-enriched protein samples. The results showed that, compared with RGS and SDC, two commonly used enzyme- and MS-compatible detergents in membrane proteomics, SL was more effective for the extraction and identification of membrane proteins particularly those with strong hydrophobicity and/or multiple transmembrane domains, demonstrating that SL has high potential for the analysis of membrane proteomes.

## Methods

### Materials and Chemicals

Standard proteins (BSA, myoglobin, bacteriorhodopsin), sodium laurate (SL), sodium deoxycholate (SDC), proteomics sequencing-grade trypsin and chymotrypsin, dithiothreitol (DTT), iodoacetamide (IAA), trifluoroacetic acid (TFA), 4-(2-hydroxyethyl)-1-piperazine ethanesulfonic acid (HEPES), α-Cyano-4-hydroxycinnamic acid (CHCA), phenylmethylsulfonyl fluoride (PMSF), ethyl acetate, *N*-α-benzoyl-L-arginine ethyl ester (BAEE), *N*-α-benzoyl-L-tyrosine ethyl ester (BTEE) and sucrose were purchased from Sigma-Aldrich (St. Louis, MO, USA). Sodium 3-[(2-methyl-2-undecyl-1, 3-dioxolan-4-yl) methoxyl]- 1-propanesulfonate (trade name *Rapi*Gest SF, RGS) was obtained from Waters Corp. (Milford, MA, USA). Acrylamide, bisacrylamide, glycine, Tris, and SDS were from Amresco (Solon, OH, USA). Bio-Rad RC DC protein assay kit was from Bio-Rad Laboratories (Hercules, CA, USA). Ultrapure 18.2-MΩ water was obtained from a Millipore Milli-Q system (Bedford, MA, USA). All other reagents were domestic products of highest grade available. Rats were purchased from Medical Academy of Central south university (Changsha, Hunan, China).

### Preparation of Rat Liver Plasma Membrane (PM) Fraction

Preparation of rat liver PM fraction was performed according to the method described [13]–[15]. Briefly, rats were anesthetized with ether and killed by decapitation after being starved for 24 h and liver blood was removed by infusing 0.9% NaCl. Liver tissues were excised and homogenized in an ice-cold solution containing 50 mM HEPES (pH 7.4), 1 mM CaCl_2_ and 0.5 mM PMSF. The mixture was filtered and centrifuged at 1500×g for 10 min at 4°C. The pellet was mixed with 50% sucrose, on the top of which 44% and 42.3% sucrose solutions were carefully layered sequentially. After centrifugation at 100000×g for 3 h at 4°C (SW-28 rotor, Beckman), the crude PM at the top of 42.3% sucrose was collected and washed twice with 1 mM sodium bicarbonate solution. For further purification, the yielding pellets were mixed with 50% sucrose, and 30% sucrose was added to the mixture until the final concentration of sucrose was 44%, over which 42.8, 42.3, 41.8, 41.0, 39.0 and 37.0% sucrose were sequentially layered. After centrifugation at 100000×g for 5 h at 4°C (SW-28 rotor, Beckman), the floating PM between 41.0% and 37.0% was collected, washed twice with the homogenization buffer and 50 mM NH_4_HCO_3_, and then stored at –80°C until use.

All the experiments were approved by the Committee on the Use and Care of Animals at the Hunan Province, P. R. China and were conducted in accordance with the guidelines established by the Committee.

### Comparison of the Abilities of Different Detergents to Lyse Membranes and Extract Membrane Proteins

Protein content of the PM fraction prepared from rat liver was quantified by a Bio-Rad RC DC protein assay kit with BSA as a standard. Equal aliquots of the PM fraction (each containing 50 µg of total protein) were solubilized with 1.0% SDS, 1.0% SL, 1.0% RGS, 1.0% SDC or 5.0% SDC for 30 min, and then sonicated twice in a water bath each for 10 min. After centrifugation at 12000 rpm for 10 min, the supernatants were collected and concentrated in a Speed-Vac (Labconco, Kansas, MO, USA), respectively. Both the supernatants and the pellets were subjected to SDS-PAGE analysis. Briefly, all the supernatant and pellet samples were separately dissolved by loading buffer containing 4.0% SDS and 10 mM DTT. After heating at 90°C for 10 min, all samples were separated by SDS-PAGE in parallel lanes on a 4.8% stacking gel and a 10% separation gel (thickness 1 mm, containing 10 wells), as described by Laemmli with minor changes [16]. After electrophoresis, the proteins were stained with Coomassie G-250 for comparative evaluation of the abilities of the detergents to lyse membranes and extract the proteins.

### Enzyme Activity Measurements

*N*-α-benzoyl-L-arginine ethyl ester (BAEE) was used as trypsin substrate. Trypsin activity was measured by on-line monitoring the hydrolytic rate of BAEE into a UV-active product *N*-α-benzoyl-L-arginine (BA) using a UV spectrophotometer (UV 2300, Techcomp, Shanghai, China) at 253 nm, according to the method previously described by Schwert et al [12], [17]. Briefly, trypsin activity assays were performed in buffered solutions containing 0%, 0.1%, 0.5%, 1.0% SL and 0.1% SDS, respectively. For each assay, trypsin (1∶20 w/w enzyme-to-substrate ratio) was added to BAEE that was dissolved in 25 mM Tris buffer. UV absorbance of the reaction solutions was recorded in 1.0 min intervals, and used to calculate the slope of ΔA253 nm versus time, which defines the trypsin activity. Changes in trypsin activity caused by the addition of different concentrations of detergents were measured by normalizing the BAEE hydrolytic rate against that of a normal sample (no detergent added). At the same time, the effect of the detergents on the activity of chymotrypsin was also investigated. The method used was similar to that for trypsin. The main differences were using *N*-α-benzoyl-L-tyrosine ethyl ester (BTEE) in stead of BAEE as the substrate and recording UV absorbance of the UV-active product *N*-Benzoyl-L-Tyrosine (BT) at 256 nm [12], [17].

### Removal of SL from Digested Sample with Phase-transfer Method

SL removal by phase-transfer method was performed according to the reported procedure for SDC removal [9], [13] with slight modification. Briefly, an equal volume of ethyl acetate was added to the digested solution, and the mixture was acidified with diluted TFA (0.5%) to about pH 2. The mixture was shaken for 1 min and then centrifuged at 15700×g for 5 min to accelerate phase separation. The SL-containing organic phase was removed and the aqueous phase was collected, concentrated in a Speed-Vac (Labconco, Kansas, MO, USA) and stored at −20°C for further analysis.

### Analysis of SL Compatibility with Mass Spectrometry

In order to illustrate the completeness of SL removal by phase-transfer method and investigate the effect of residual SL, if any, on subsequent MS analysis, six aliquots of digested peptides of BSA (100 fmol each) were separately used for following treatments prior to MALDI TOF MS analysis: (A) The sample, as a control, was directly used for the analysis; (B) and (C) SL was added at concentrations of 0.1% and 0.5% (final concentration), respectively, and the mixtures were used for MS analysis without phase-transfer treatment; (D) and (E) SL was added at 0.1% and 0.5% (final concentration), respectively, followed by SL removal with phase-transfer method. The evaluation of SL compatibility with mass spectrometry was made by comparison of the number and S/N ratio of peptide ions in the MS spectra of the six samples. The information of peptides was obtained by using a MALDI-TOF-TOF mass spectrometer (UltraFlex, Bruker Daltonics, Bremen, Germany) equipped with nitrogen laser (337 nm) and operated in reflector/delay extraction mode.

In-solution Digestion of Standard ProteinsStandard protein mixture was composed of three proteins: myoglobin (MW: 17 kDa), bacteriorhodopsin (MW: 26 kDa), BSA (MW: 66 kDa). The mixture contained equal amount (5 µg) of each protein and was dissolved in 50 mM NH_4_HCO_3_ (pH 7.8), 1.0% SL/50 mM NH_4_HCO_3_ (diluted to 0.1% SL prior to digestion), 1.0% RGS/50 mM NH_4_HCO_3_ (diluted to 0.1% RGS prior to digestion) or 1.0% SDC/50 mM NH_4_HCO_3_ solution. The protein mixtures with a total concentration of 0.5 µg/µL were digested with trypsin for 12 h at 37°C (enzyme-to-protein ratio 1∶50).

### Extraction and In-solution Digestion of the Proteins from Rat Liver PM Fraction

Extraction and digestion of the proteins from PM-enriched fraction were carried out according to the procedure described in our previous paper [12], [13]. For comparison, three aliquots of membrane fractions (each containing 20 µg of total protein) were separately solubilized in 1.0% SL/50 mM NH_4_HCO_3_ (diluted to 0.1% SL prior to digestion), 1.0% RGS/50 mM NH_4_HCO_3_ (diluted to 0.1% RGS prior to digestion) or 1.0% SDC/50 mM NH_4_HCO_3_ solution. Proteins were reduced with 5 mM DTT for 60 min, and then alkylated in the dark with 25 mM IAA for 45 min at room temperature. After the final concentration of proteins was adjusted to 0.2 µg/µL, trypsin was added at an enzyme-to-protein ratio of 1∶50 and incubated at 37°C for 16 h. Following digestion, all reaction mixtures were acidified with diluted TFA to stop the proteolysis. After acidification, the digests in RGS and SDC solution were centrifuged for 10 min at 15700×g to remove the yielding insoluble materials [10], [12]. The supernatants were collected, concentrated in a Speed-Vac (Labconco, Kansas, MO, USA) and stored at −20°C for further use. The digests in SL solution were treated by phase-transfer method for SL removal [13].

### CapLC-MS/MS Analysis of the Proteins in Standard Mixture and Rat Liver PM Fraction

Tryptic digests of standard protein mixture and the proteins in rat liver PM preparations were analyzed by automated capillary liquid chromatography-tandem mass spectrometry (CapLC-MS/MS). Liquid chromatography was performed using an automated Agilent 1200 LC system (Agilent Technologies, Waldbronn, Germany) with an autosampler equipped with a 40 µL capillary loop. Samples were desalted and preconcentrated at a flow rate of 20 µL/min on a short C18 precolumn Zorbax SB (500 µm i.d., 3.5 cm, Agilent) connected in the front of an analytical capillary column. For the separation with the C18 PepMap column (180 µm i.d., 15 cm, LC-Packings, Amsterdam, The Netherlands), a flow rate of 3 µL/min was generated by a cap-flow splitter cartridge (3/500) and an initial pump flow rate of 500 µL/min. Each sample injection volume in all experiments was 30 µL. For the chromatography, the following solvents were used: solvent A (99.9% H_2_O, 0.1% FA), solvent B (99.9% ACN, 0.1% FA). The elution gradients used for separation of tryptic digests of standard protein mixture and those of rat liver PM proteins were respectively from 5% to 33% B in 35 min and 155 min, 33% to 80% B in 8 min and 15 min, followed by 80% B for 8 min and 10 min, then by 5% B in 9 min and 10 min. The chromatography system was directly coupled to a 3D high-capacity ion trap (HCTultra™, Bruker Daltonics, Bremen, Germany) mass spectrometer with an electrospray ionization source. Instruments were controlled using Chemstation B01 (Agilent) and EsquireControl™ 6.0 (Bruker Daltonics) software. Nebulizer pressure was 10 psi. Flow rate of drying gas was 5 L/min. Temperature of drying gas was 300°C. Capillary voltage was 4000 V. Full MS scan mode was standard-enhanced (m/z 350 to 1600). The four most abundant ions detected in each MS scan were selected for collision-induced dissociation with 1.05 V collision energy. The peptides were analyzed using the data-dependent MS/MS mode over the m/z range of 200–2000.

### Data Processing and Bioinformatics

Raw spectral data were processed and Mascot compatible mgf files were created using DataAnalysis™ 3.4 software (Bruker Daltonics) with the following parameters: compounds threshold 10000, maximum number of compounds 100000, retention time windows were 1.0 min. MS/MS ion searches were performed using Mascot™ 2.2 software (Matrixscience, London, UK). The MSDB database and the international protein index (IPI) rat database (IPI_rat_v3.64) downloaded as FASTA-formatted sequences were used for protein identification. Search parameters were set as follows: enzyme, trypsin; allowance of up to one missed cleavage site; mass tolerance, 1.2 Da and MS/MS mass tolerance, 0.6 Da; fixed modification, carbamidomethylation (at Cys); variable modification, oxidation (at Met). Proteins were identified on the basis of two or more peptides whose ions scores exceeded the threshold, *P*<0.05, which indicated identification at the 95% confidence level. If proteins were identified by a single peptide, the spectrum was manually inspected. For a protein to be confirmed, the assignment had to be based on four or more y-or b-series ions (e.g., y4, y5, y6, y7). A perl script was written in-house to parse Mascot output files (html files) into XML files suitable for subsequent data analysis. All identified proteins in liver PM preparation had an IPI database accession number and many of these proteins have assigned Gene Ontology (GO) numbers [18], which were used to retrieve the protein information in the database. The average hydropathy expressed as GRAVY value [19] for identified proteins was calculated using the ProtParam software available at http://cn.expasy.org. Predictions for putative transmembrane domains (TMDs) in all identified proteins were carried out using the transmembrane hidden markov model (TMHMM) algorithm [20], available at http://www.cbs.dtu.dk/services/TMHMM. False positive rates were evaluated by using the reversed sequence databases search strategy [21].

## Results and Discussion

### Structure Analysis of Detergents

In membrane proteomic studies, the extraction and solubilization of membrane proteins is a critical step, which heavily affects the analytical results of such kind of proteins. SDS is defined as the most efficient detergent for extracting, solubilizing and denaturing membrane proteins as well as preventing protein aggregation [9], [22]. The property of SDS is dependent on its chemical structure: its long, flexible hydrocarbon tail forms hydrophobic interactions with polypeptide chains-irrespective of amino acid identity or sequence, breaking existing membrane-protein interactions [8]. However, when SDS is applied to solution-based shotgun proteome analyses, its shortcomings are also obvious: (1) it may severely hinder enzyme activity at slight higher concentrations [10], [13] and (2) is difficult to remove before subsequent analyses, which is quite problematic because SDS can interfere with LC and severely suppress ionization of peptides by MALDI and ESI [23]–[25]. Hence, when the SDS-assisted strategy is employed, the total efficiency of membrane protein identification is usually not desired. Our previous studies have demonstrated that SDC could be perfectly compatible with protease activity and LC-MS/MS analysis, thus being suited for the shotgun analysis of membrane proteomes [12], [26]. However, during the process of experiments, we also found that SDC had limitations in lysing membranes and there was always a few of residual membranes left after solubilization with SDC. The reason for this is that it is a steroidal compound, having a polar face and an apolar face rather than a linear amphaphilic molecule with distinct “head” and “tail” groups. Starting with comparative analysis of the structures of SDS and SDC, we finally found that sodium laurate (SL) has a long hydrophobic hydrocarbon chain similar to that of SDS and a hydrophilic head (a carboxyl group) similar to that of SDC (Figure S1). In other words, SL has the tail structure of SDS and the head structure of SDC. We speculated that this characteristic of SL may make it become an even more suitable additive that not only efficiently extracts the membrane proteins but also is well compatible with subsequent proteolysis and LC-MS/MS analysis in membrane proteomics.

### Membrane Protein Extraction Efficiency

**Figure 1:**
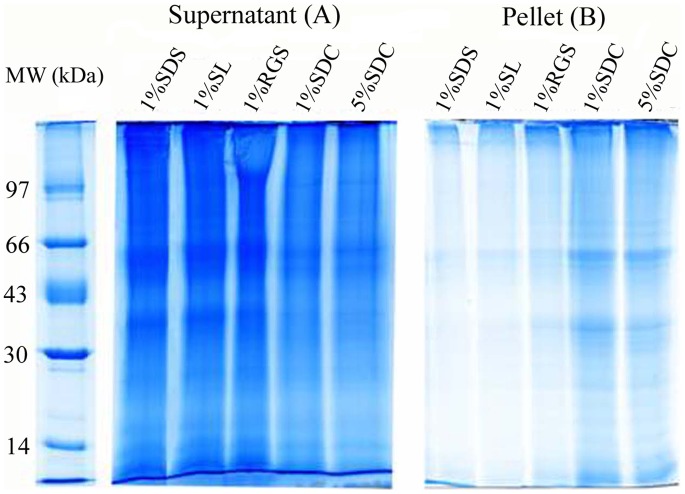
Comparison of the abilities of four different detergents to extract membrane proteins. (A) SDS-PAGE analysis of the proteins in the supernatants of PM-enriched fraction dissolved by different detergents followed by centrifugation; (B) SDS-PAGE analysis of the proteins remaining in the pellets after detergent extraction. For SDS-PAGE analysis, the pellets were dissolved by loading buffer containing 4% SDS.

For evaluating the ability of SL to disrupt membranes and extract membrane proteins, it was compared with several commonly used detergents (SDS, SDC and RGS). The SDS-PAGE results of the supernatants and pellets after dissolving membranes using the four different detergents are shown in Figure 1. It can be found from the Figure 1A that the staining intensity of proteins in the supernatants after dissolving membrane by 1.0% SL (lane 1% SL) is comparable to that by 1.0% SDS (lane 1% SDS), slightly higher than that by 1.0% RGS, and much higher than those by 1.0% and 5.0% SDC, indicating that SL is comparable to SDS with regard to their ability to disrupt the membranes and extract membrane proteins. In addition, the protein contents in the supernatants were also quantified with a Bio-Rad RC DC protein assay kit and the same conclusion was found (data not shown). The SDS-PAGE images of the pellets are shown in Figure 1B. Comparatively, the staining intensity of proteins in lane 1% SL is also comparable to that in lane 1% SDS, and is lower than those in other lanes, suggesting that, after extraction with 1.0% SL and 1.0%SDS, the residual proteins in pellets, which represent the lost proteins, were the fewest. The conclusion drawn from the pellet analysis is in agreement with that from the supernatant analysis. Through the above analyses, we believe that SL is also a strong detergent and has a high potential in membrane proteome analysis.

### Effect of SL on Protease Activity

**Figure 2:**
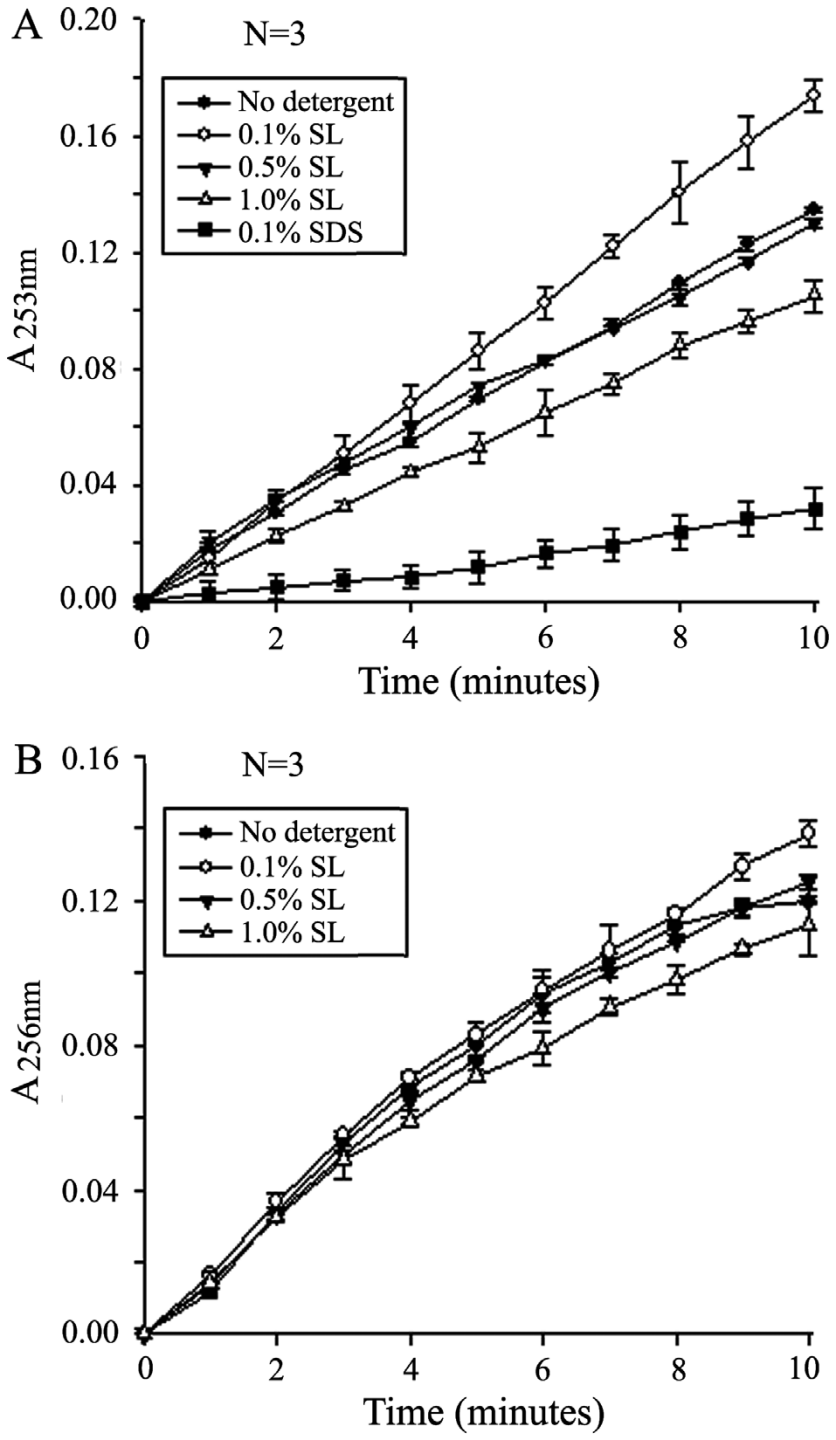
Enzyme activity measured in the presence of different concentrations of SL. (A) Trypsin. BAEE was hydrolyzed by trypsin to UV-absorbing product BA. Trypsin activity was measured as a slope of the UV absorbance change at *A*_253 nm_. (B) Chymotrypsin. BTEE was hydrolyzed by chymotrypsin to UV-absorbing product BT. Chymotrypsin activity was measured as a slope of the UV absorbance change at *A*_256 nm_. Data were averaged from three measurements.

Trypsin activity was determined as the hydrolytic rate of *N*-α-benzoyl-L-arginine ethyl ester (BAEE) into a UV-active product *N*-α-benzoyl-L-arginine (BA) in the presence of different concentrations of SL (Figure 2A). Because the reaction follows zero-order kinetics (enzyme saturation), the absorbance of the product increases in a linear fashion until all the substrate is converted into product [12], [17]. The reaction during initial stage almost completely followed zero-order kinetics because the substrate concentration was high enough. Hence, in our experiment, we recorded the data from the first 10 min of reaction, which was used to draw the curve of ΔA_253nm_ versus time. The slope of the curve in initial stage was defined as the relative trypsin activity. Compared with the control experiment (no SL added), little decrease in trypsin activity was observed in the presence of 0.5% SL; 1.0% SL only moderately decreased the trypsin activity (about 20%); When the concentration of SL was 0.1%, the trypsin activity was enhanced. Meanwhile, we also measured the effect of 0.1% SDS on the trypsin activity and found that SDS at this concentration severely inhibited trypsin activity by about 80%, which is in agreement with the result reported by Yu et al [10].

In addition, we also measured chymotrypsin activity in the presence of different concentrations of SL (Figure 2B). The sites of chymotryptic cleavage are different from those of tryptic cleavage, which can be used to further confirm the effect of SL on protease activity. The results in Figure 2B are similar to those in Figure 2A. The slight difference is that the amplitudes of changes in enhancing or inhibiting chymotrypsin activity by different concentrations of SL are smaller than those of changes in trypsin activity, indicating that the effect of SL on chymotrypsin is smaller than on trypsin. It can be drawn from the above results that SL not only does not obviously inhibit the proteases at the concentrations up to 0.5% but also can enhance the enzyme activity at lower concentrations, which present the attractive prospect of its application in membrane proteomics.

### SL Compatibility with Mass Spectrometry

**Figure 3:**
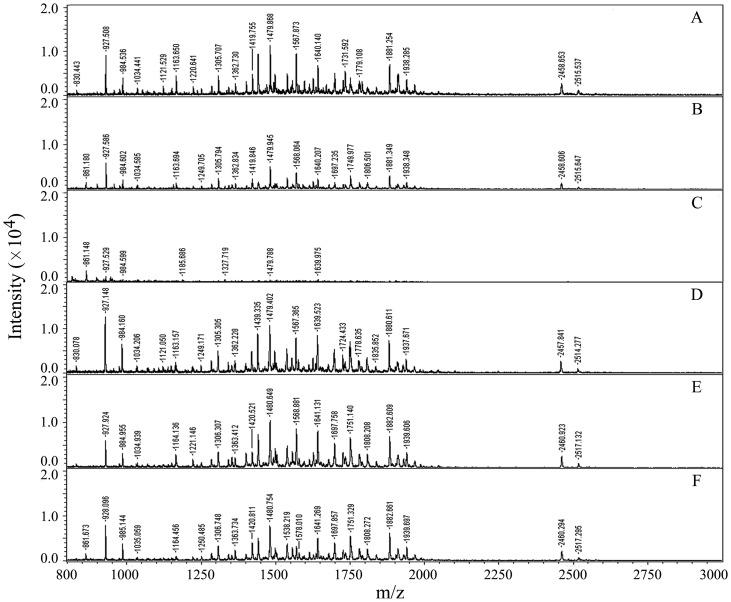
MALDI-TOF MS spectra of tryptic digests of BSA. (A) mixed with matrix (1∶1) without addition of SL; (B and C) mixed with matrix (1∶1) after addition of SL (the final concentrations were 0.1% and 0.5%, respectively); (D–F) mixed with matrix (1∶1) after addition of SL (the final concentrations were 0.1%, 0.5% and 1.0%, respectively) and then removing it by acidification and phase-transfer method.

Many detergents such as SDS are known to suppress MS ion signals of analytes and give a high MS background even at trace concentrations [27], which severely make interference with MS analysis and protein identification. It was previously shown that SDC was compatible with mass spectrometry, because it precipitated under low-pH conditions and was easily removed from sample solution by centrifugation or phase-transfer after acidification prior to mass spectrometric analysis [12], [13]. SL was similar to SDC in that it also could generate the insoluble material (lauric acid) by acidification. Because the density of lauric acid was lower than that of water, centrifugation is not suited for the removal of SL. In the present experiment, we used the phase-transfer method to remove SL as it was applied to SDC removal [9], [13]. In order to illustrate the efficiency of this method in SL removal, the six samples of digested BSA treated with different methods were analyzed by MALDI-TOF MS (Figure 3). It was found that, compared with the control (Figure 3A), the ion signals of peptides without removal of the added 0.1%SL were obviously suppressed (Figure 3B). When the concentration of SL was increased up to 0.5%, the ion signals of peptides were almost completely suppressed and only a few of ion peaks presented in the mass spectrum (Figure 3C), suggesting that SL had an inhibitory effect on the ionization of peptides in mass spectrometric analysis. However, after the SL was removed from the digest with phase-transfer method, the number and S/N ratio of peptide ions in the spectra were not significantly affected (Figure 3D and 3E). These results showed that, although the presence of SL in the sample could suppress the ionization of peptides, it could be easily removed with phase-transfer following acidification. The remaining SL, if any, could hardly make interference with MS analysis.

### Analysis of Standard Protein Mixture

In order to evaluate the effect of SL on the enzymolysis and thus the identification of proteins especially hydrophobic membrane proteins, a protein mixture of BSA, myoglobin and the typical transmembrane protein bacteriorhodopsin was prepared as a simulative natural protein mixture. These proteins have different susceptibilities toward proteolysis. BSA is a soluble protein and is amenable to proteolytic digestion. In contrast, myoglobin is a compact globular protein and is known to be severely resistant to proteolytic digestion [12], and bacteriorhodopsin as a membrane protein with seven transmembrane domains is difficult to dissolve and digest in pure aqueous buffers [24]. After the protein mixture was digested with trypsin in the presence and absence of RGS, SDC and SL, respectively, the resulting peptide mixtures were separately analyzed by LC-MS/MS. The number of unique peptides and sequence coverage for each protein identified with Mascot search are listed in Table 1. It could be found that BSA was almost equally efficiently identified under all the four digestion conditions. However, in 50 mM NH_4_HCO_3_ buffer, myoglobin was identified with lower number of unique peptides and sequence coverage, whereas bacterirohodopsin was not identified at all. The addition of detergents was obviously helpful for the identification of the two proteins. Comparatively, the identification efficiency of proteins digested in 0.1% SL was the highest in terms of the unique peptide number and sequence coverage for the identified proteins. Compared with the other digestion methods, SL-assisted digestion increased the number of unique peptides of proteins on average by 18.2%, and increased the sequence coverage on average by 19.7%. Although bacteriorhodopsin is an insoluble and proteolytically resistant protein, it was unambiguously identified from the mixture digested in the presence of 0.1% SL with higher unique peptide number (4 peptides) and sequence coverage (21%) than in the presence of SDC (2 peptides and 12% coverage) and RGS (3 peptides and 16% coverage).

**Table 1:** Comparative analysis of standard protein mixture identified with CapLC-MS/MS after digested by four different strategies.

Proteins	50 mM NH_4_HCO_3_		1.0%–0.1% RGS		1.0% SD		1.0%–0.1% SL		
	UP	SC (%)	UP	SC (%)	UP	SC (%)	UP	SC (%)	
Bacteriorhodoposin	0	0	3	16	2	12	4	21	
Myoglobin	5	46	8	62	8	64	10	69	
BSA	32	54	34	58	35	59	36	58	

Note: 1.0%–0.1% RGS (or SL) represents that the proteins were dissolved in 1.0% RGS (or SL) and then diluted to 0.1% for digestion. 50 mM NH_4_HCO_3_ or 1.0% SDC represents that the proteins were dissolved and digested in 50 mM NH_4_HCO_3_ or 1.0% SDC. UP, unique peptide; SC, sequence coverage.

### Identification of Proteins in Rat Liver PM-enriched Fraction

**Figure 4:**
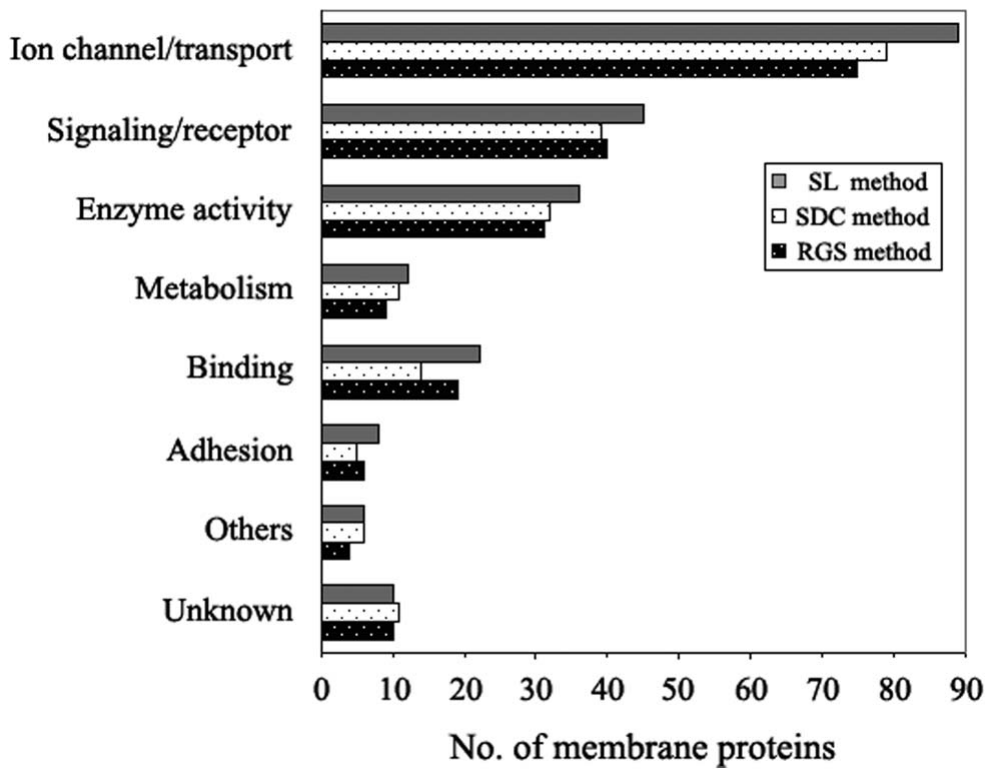
Comparison of the distributions of membrane proteins based on their function annotations identified by the three different detergent-assisted methods. The merged results from triplicate analysis in each method were used for comparison.

For further evaluating the ability of SL to improve the extraction, enzymolysis and identification of membrane proteins, a comparative study involving different additives was made on the tryptic digestion and identification of proteins in rat liver PM-enriched fraction, a real protein mixture, including both hydrophobic integral membrane proteins and hydrophilic peripheral membrane proteins as well as some other proteins. After the preparation was solubilized and digested in RGS-, SDC- and SL-assisted methods, respectively, the digests were analyzed with CapLC-MS/MSfollowed by database searches. The false-positive rate was evaluated as below 3% by using the reversed sequence databases search strategy [21]. After removal of the false-positive results, a total of 398, 410 and 465 proteins were identified from the rat liver PM-enriched fraction based on the above three different digestion methods, respectively (Table 2 and Table S1). Compared with RGS- and SDC- assisted methods, SL-assisted method could get better results evaluated on both protein and peptide levels. Not only the numbers of total proteins and peptides identified were increased on average by 15.1% and 17.2%, but also the numbers of total membrane proteins and integral membrane proteins were increased on average by 16.6% and 15.8%, respectively. In addition, the average number of unique peptides per protein in SL-assisted method (3.72) was higher than that in RGS- or SDC-assisted method (3.68 or 3.62) (Table 2), suggesting that the identification of proteins in SL-assisted method has even higher sequence coverage and thus the reliability. Then we categorized the GO function annotations of the identified membrane proteins, though the classification was not strict due to the fact that a protein usually has multiple functions. As shown in Figure 4, of identified membrane proteins in the three methods (RGS, SDC and SL), 75 (38.7%), 79 (40.1%), 89 (39.1%) are ion channel or transport proteins, respectively; 40 (20.6%), 39 (19.7%), 45 (19.7%) are receptor or signaling transduction proteins; 40 (20.6%), 43 (21.8%), 48 (21.1%) are enzymes and the proteins involved in metabolism; 29 (14.9%), 25 (12.8%), 36 (15.8%) are the proteins involved in the binding, cell adhesion and other cellular functions; In addition, 10 (5.2%), 11 (5.6%), 10 (4.3%) proteins have no function annotation and were classified into the group “unknown”. From the data analysis we knew that the proteins with ion channel/transport and signaling/receptor functions, which were generally considered to be low abundant and hydrophobic, accounted for the largest group, The proteins involved in catalytic activity and metabolism make up the second largest group, suggesting that the membrane proteome plays important roles in the substance exchange, signal transduction and metabolism in the cell. Moreover, about 12.0% of the identified membrane proteins involve cell recognition, adhesion and binding, reflecting the importance of membrane proteins in cell communication and function integration. Importantly, in most groups, SL method led to more proteins to be identified compared with other two methods, indicating that it has superiority in the identification of membrane proteins. In the above analyses, the distributions of identified proteins were displayed as merged data from triplicate analysis. Meanwhile, the statistical analysis of identified proteins using average values and standard deviations from the triplicates was also made (Figure S2A in Figure S2), which showed that more membrane proteins were reproducibly identified with the SL method and the triplicate data were also internally consistent. T-test showed that the membrane proteins identified in SL-assisted method were significantly more than that identified in RGS-assisted method (p<0.05) and, however, the difference between SL-method and SDC-method was not significant (p>0.05) (Figure S2C in Figure S2).

**Table 2:** Comparative analysis of proteins and their matching peptides identified by three strategies from rat liver plasma membrane-enriched fraction.

Categorization	Strategies		
	RGS	SDC	SL
All identifiedproteins	398	410	465
All identifiedpeptides	1466	1485	1729
Peptides/proteins	3.68	3.62	3.72
Membraneproteins	194	197	228
Hydrophobicproteins	111	111	132
Hydrophobicpeptides	731	736	839
Proteins with 1 ormore predicted TMDs	128	131	150
Proteins with more than 2 predicted TMDs	49	48	62

The data merged from triplicate analysis were used for comparison.

**Figure 5:**
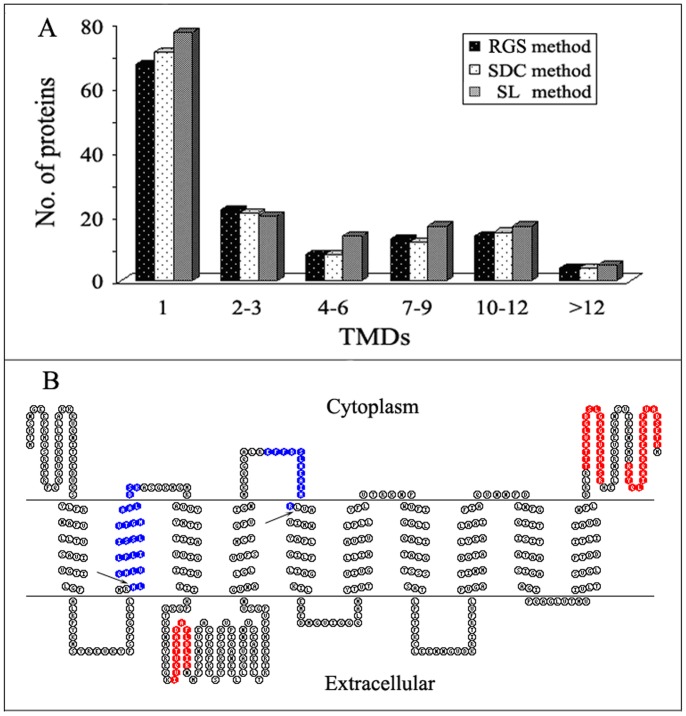
Comparison of the transmembrane proteins identified from rat liver PM-enriched fraction in three different detergent-assisted methods. (A) Comparison of the distributions of all the identified integral membrane proteins as a function of TMDs. The data merged from triplicate analysis were used for comparison. (B) Comparison of sequence coverages of the integral membrane protein isoform GLAST-1 of excitatory amino acid transporter 1 (IPI00324377) identified by RGS, SDC or SL method. The sequence coverages were visualized with the TOPO2 transmembrane protein graphics program. The tryptic peptides identified by all three methods were in red, and those newly identified only by SL method were in blue, whose two tryptic cleavage sites inside the TMDs are indicated with black arrows.

Generally, integral membrane proteins are more difficult to analyze than other proteins. Because they are usually integrated into lipid bilayer and are tramembrane proteins containing one or multiple transmembrane domains (TMDs) that are commonly highly hydrophobic, it is constantly a challenge for the identification of these proteins in membrane protein researches. In the present study, we especially evaluated and compared the effects of SL, RGS and SDC on the identification of these challenging proteins in natural PM-enriched sample by analyzing the TMDs of the identified integral membrane proteins. The numbers of transmembrane proteins identified based on digestion in the presence of RGS, SDC and SL were 128, 131 and 150, respectively. The detail distribution profiles of identified transmembrane proteinsare showed in Figure 5A. The distributions of identified transmembrane proteins in the figure are displayed as merged data from triplicate analysis whereas the statistical analyses of the distributions using average values and standard deviations from the triplicates are shown in Figures S2B and S2C in Figure S2, from which it could be found that SL-method was significantly more efficient than RGS- and SDC-assisted methods in the identification of integral membrane proteins (p<0.05).

**Figure 6:**
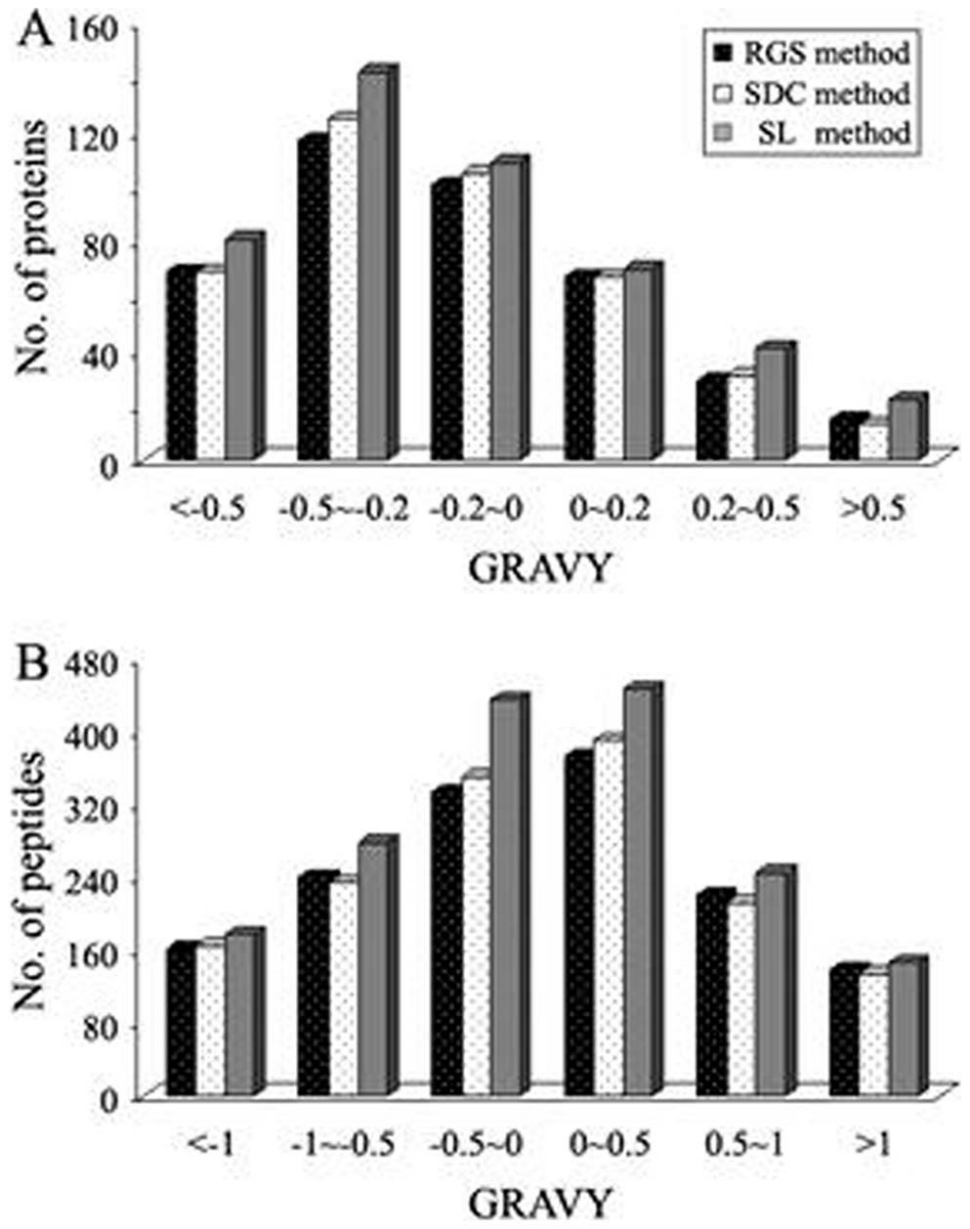
Comparison of the distributions of proteins and their matching peptides identified based on the three different digestion methods. (A) Distribution of identified proteins as a function of the calculated grand average of hydropathy (GRAVY) values. (B) Distribution of identified peptides as a function of the calculated grand average of hydropathy (GRAVY) values. The data merged from triplicate analysis were used for comparison.

It could be found from Figure 5A that, of the identified transmembrane proteins, the proteins with 1 TMD accounted for a large proportion. Further inspection indicated that the numbers of transmembrane proteins with 1 and greater than 3 TMDs identified in SL-assisted method were more than those in the other two methods. The superiority of SL exhibited in the identification of membrane proteins especially those with multiple TMDs suggests that it is even more suitable for the analysis of membrane proteomes. For example, as shown in Table S1, NADH dehydrogenase subunit 5 (IPI00734731) with 15 TMDs was identified based on three unique peptides only in the SL-assisted method. This protein was reported to have been identified based on one unique peptide as the membrane protein with the maximum number of TMDs in all proteins identified in trifluoroethanol (TFE)-assisted method [28]. Another example of identified transmembrane protein is showed in Figure 5B. When the protein isoform GLAST-1 of excitatory amino acid transporter 1 (IPI00324377) with 10 TMDs was identified, the number of matching peptides (marked with red color in Figure 5B) and the sequence coverage were 3 and 10%, respectively, in both RGS- and SDC-assisted methods. However, when it was identified in SL-assisted method, the number of matching peptides (marked with red and blue color) and the sequence coverage were increased to 5 and 17%, respectively. It is worthy noting that the two peptides newly identified in SL-assisted method involve two cleavage sites inside TMDs (indicated with arrows in Figure 5B), which is usually difficult to cleave with trypsin under common experimental conditions.Because the hydrophobicity of protein or peptide is one of the crucial factors influencing the efficiency of membrane protein digestion and identification, as demonstrated by the above analyses, we also comparatively evaluated the actions of the used different additives on the identification of peptides and proteins based on the GRAVY values. At present, it is generally accepted that proteins with positive GRAVY value are classified as hydrophobic proteins, negative as hydrophilic [19]. As shown in Table 2, Tables S1 and S2, SL method gave rise to the highest numbers of identified hydrophobic peptides and proteins (839 and 132), followed sequentially by SDC method (736 and 111), and RGS method (731 and 111). Then we categorized the identified proteins and peptides into six groups according to their GRAVY values (Fig. 6). Compared with other two methods, SL method led to more proteins and peptides to be identified in all groups especially the groups of high GRAVY values, where some proteins or peptides with very high GRAVY values were identified only in SL method, such as the protein solute carrier family 22 member 18 (IPI00464615) and the peptide IIVIM that have the highest GRAVY value of 0.93 and 3.92, respectively, in all the identified proteins and their matching peptides (Supplementary Tables S1 and S2). These experimental results demonstrated that SL-assisted method had superiority in the identification of hydrophobic peptides and proteins in the natural protein mixture, which was speculated to be due to the ability of SL to improve the solubilization and digestion of the proteins.

### Conclusions

SL as a strong surfactant can be used at high concentrations to extract and solubilize the membrane proteins particularly integral membrane proteins. Its action effect is comparable to that of SDS, and superior to those of RGS and SDC. Enzyme activity assay demonstrated that, when the concentration of SL was 0.1%, the trypsin activity could be enhanced, and little decrease in trypsin activity was observed when SL concentration was up to 0.5%. Even 1.0% SL only moderately decreased the trypsin activity (by about 20%). The effect of SL on chymotrypsin activity was found to be even smaller. In addition, SL could be easily removed from sample solution with phase-transfer method following acidification, and the remaining SL, if any, could hardly make interference with MS analysis. Therefore, SL can be classified as a new protease- and MS-compatible detergent. The application of SL to the analyses of standard and rat liver PM-enriched protein samples showed that, compared with RGS and SDC, another two commonly used protease- and MS-compatible detergents, SL could more efficiently improve the identification of membrane proteins particularly the integral membrane proteins with multiple transmembrane domains and/or high hydrophobicity, which may be attributed to its strong ability to sollubilize the membrane proteins and good compatibility with protease activity as well as mass spectrometry.

## Supporting Information

Figure S1 **The structures of three detergents sodium dodecyl sulfate (SDS), sodim deoxycholate (SDC) and sodium laurate (SL).**
(DOC)

Figure S2 Figure S2A shows the statistical analysis of the distributions of membrane proteins identified from rat liver PM-enriched fraction based on their function annotations, using average values and standard deviations from triplicate analysis in each method for comparison. Figure S2B shows the statistical analysis of the distributions of transmembrane proteins identified from rat liver PM-enriched fraction as a function of TMDs, using average values and standard deviations from triplicate analysis in each method for comparison. Figure S2C shows the statistical analysis of the differences of the three methods in the identification of membrane proteins and integral membrane proteins.
(DOC)

Table S1 **The information on all the proteins identified from the rat liver PM-enriched fraction by three different methods.**
(XLS)

Table S2 **The information on the non-redundant peptides identified by the three different methods.**
(XLS)
