# Supplementary material for: Sodium Laurate, a Novel Protease- and Mass Spectrometry-Compatible Detergent for Mass Spectrometry-Based Membrane Proteomics
Source: PLoS One. 2013 Mar 28;8(3):e59779. doi: 10.1371/journal.pone.0059779 (PMC3610932; doi:10.1371/journal.pone.0059779)
Supplement: Figure S1 — The structures of three detergents sodium dodecyl sulfate (SDS), sodim deoxycholate (SDC) and sodium laurate (SL). (DOC) [file pone.0059779.s001.doc]

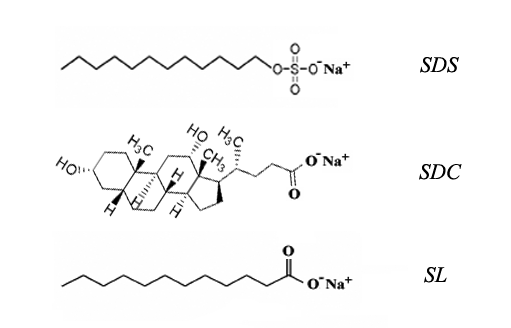


**Supplementary Figure S1.** The structures of three detergents. SDS, sodium dodecyl sulfate; SDC, sodim deoxycholate; SL, sodium laurate.
