# Supplementary material for: Sodium Laurate, a Novel Protease- and Mass Spectrometry-Compatible Detergent for Mass Spectrometry-Based Membrane Proteomics
Source: PLoS One. 2013 Mar 28;8(3):e59779. doi: 10.1371/journal.pone.0059779 (PMC3610932; doi:10.1371/journal.pone.0059779)
Supplement: Figure S2 — A shows the statistical analysis of the distributions of membrane proteins identified from rat liver PM-enriched fraction based on their function annotations, using average values and standard deviations from triplicate analysis in each method for comparison. Figure S2B shows the statistical analysis of the distributions of transmembrane proteins identified from rat liver PM-enriched fraction as a function of TMDs, using average values and standard deviations from triplicate analysis in each method for comparison. Figure S2C shows the statistical analysis of the differences of the three methods in the identification of membrane proteins and integral membrane proteins. (DOC) [file pone.0059779.s002.doc]

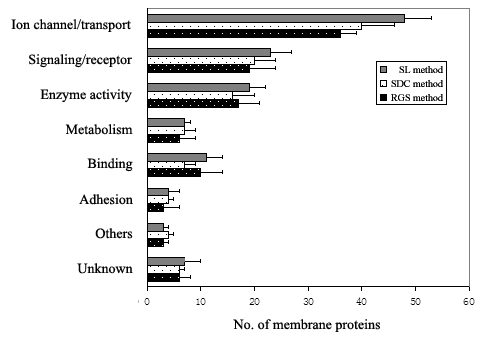


**Supplementary Figure S2A.** Statistical analysis of the distributions of identified membrane proteins based on their function annotations, using average values and standard deviations from triplicate analysis in each method for comparison.


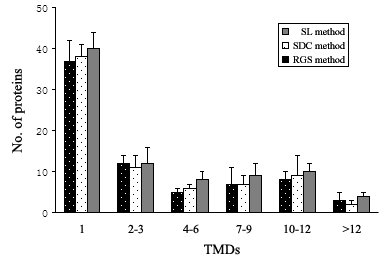


**Supplementary Figure S2B.** Statistical analysis of the distributions of transmembrane proteins identified from rat liver PM-enriched fraction as a function of TMDs, using average values and standard deviations from triplicate analysis in each method for comparison.


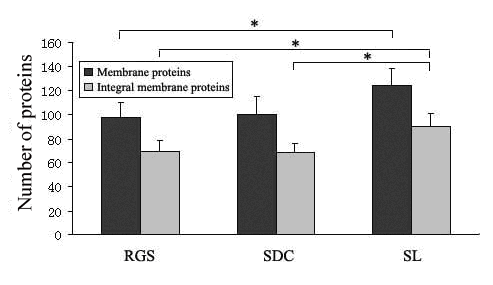


**Supplementary Figure S2C.** Statistical analysis of the differences of the three methods in the identification of membrane proteins and integral membrane proteins.

Asterisk indicates that the difference was significant (p<0.05), as determined by *t*-test.
